# Do congenital prosopagnosia and the other-race effect affect the same face recognition mechanisms?

**DOI:** 10.3389/fnhum.2014.00759

**Published:** 2014-09-29

**Authors:** Janina Esins, Johannes Schultz, Christian Wallraven, Isabelle Bülthoff

**Affiliations:** ^1^Human Perception, Cognition and Action, Max Planck Institute for Biological CyberneticsTübingen, Germany; ^2^Department of Psychology, Durham UniversityDurham, UK; ^3^Department of Brain and Cognitive Engineering, Korea UniversitySeoul, South Korea

**Keywords:** congenital prosopagnosia, other-race effect, face recognition, Asian, Caucasian

## Abstract

Congenital prosopagnosia (CP), an innate impairment in recognizing faces, as well as the other-race effect (ORE), a disadvantage in recognizing faces of foreign races, both affect face recognition abilities. Are the same face processing mechanisms affected in both situations? To investigate this question, we tested three groups of 21 participants: German congenital prosopagnosics, South Korean participants and German controls on three different tasks involving faces and objects. First we tested all participants on the Cambridge Face Memory Test in which they had to recognize Caucasian target faces in a 3-alternative-forced-choice task. German controls performed better than Koreans who performed better than prosopagnosics. In the second experiment, participants rated the similarity of Caucasian faces that differed parametrically in either features or second-order relations (configuration). Prosopagnosics were less sensitive to configuration changes than both other groups. In addition, while all groups were more sensitive to changes in features than in configuration, this difference was smaller in Koreans. In the third experiment, participants had to learn exemplars of artificial objects, natural objects, and faces and recognize them among distractors of the same category. Here prosopagnosics performed worse than participants in the other two groups only when they were tested on face stimuli. In sum, Koreans and prosopagnosic participants differed from German controls in different ways in all tests. This suggests that German congenital prosopagnosics perceive Caucasian faces differently than do Korean participants. Importantly, our results suggest that different processing impairments underlie the ORE and CP.

## Introduction

Recognizing faces is arguably the most important way to identify other humans and bears great social importance. Even though faces are a visually homogeneous object class, most humans are experts in face identification: within milliseconds we can identify a familiar face in poor lighting, after 15 years of aging, 20 pounds of weight loss, or with a different hairdo—and this is true for the several hundred acquaintances we have on average.

One explanation for this achievement is that we use “holistic processing” for faces: we integrate the different components of a face [e.g., the form and color of the features (eyes, nose, and mouth) and their configuration (i.e., spatial distances between the features)] into a whole and do not process single pieces of information individually (Maurer et al., 2002). If the retrieval of this information is disturbed, holistic processing and thus face recognition are impaired (Collishaw and Hole, 2000). Especially configural processing is considered to be one of the most important aspects of holistic processing: disturbing this process alone already strongly affects holistic processing of faces (Maurer et al., 2002).

Most humans are undoubtedly experts at every-day face recognition but this expertise can be disturbed in various ways. Two well-known phenomena in which people show impaired face recognition abilities are congenital prosopagnosia (CP) and the other-race effect (ORE).

CP is an innate impairment in face processing. People with CP often encounter social difficulties, like being considered arrogant or ignorant because they fail to recognize and greet acquaintances. Therefore, some of them tend to keep a socially withdrawn life. Presumably 2.5% of the population is affected (Kennerknecht et al., 2008). In contrast to the acquired form of prosopagnosia, which is caused by acquired brain damage, CP is inborn and there are no evident brain lesions. Also several studies found normal functional brain response to faces in fMRI studies (e.g., Avidan et al., 2005; Avidan and Behrmann, 2009) and EEG studies (e.g., Towler et al., 2012) but subtle differences in connectivity between face processing brain regions for congenital prosopagnosics compared with controls (Avidan et al., 2008). In a single case study of CP, this reduced connectivity could be enhanced by training on spatial integration of mouth and eye regions of faces. The training also had positive effects on face recognition performance but vanished after a few months (DeGutis et al., 2007).

The ORE describes the fact that we recognize faces of our own (familiar) race faster and more accurately than faces of an unfamiliar ethnicity (Meissner and Brigham, 2001). This effect (also called “cross-race bias,” “own-race advantage,” or “other-race deficit”) is a common and known phenomenon. Several models exist to explain the underlying mechanisms causing the ORE. The most common explanation is the higher level of expertise for same-race faces compared with other-race faces (Meissner and Brigham, 2001). This perceptual expertise hypothesis states that the frequent encounter and the training in individuating own-race faces leads to a greater experience in encoding the dimensions most useful to individuate faces of that race. Nevertheless, competing models exists, like the social categorization hypothesis, which states that mere social out-group categorization is sufficient to elicit a drop in face recognition performance (Bernstein et al., 2007). Another hypothesis is the categorization-individuation model which combines perceptual experience, social categorization and motivated individuation (discrimination among individuals within a racial group which requires attending to face-identity characteristics rather than to category-diagnostic characteristics), all three of which co-act and generate the ORE (Hugenberg et al., 2010). The underlying mechanisms are not clear yet, but it has been shown that the ORE can be overcome by training, but only for the trained faces (McKone et al., 2007).

As nearly everyone has experienced the ORE, it is sometimes cited as an example by congenital prosopagnosics when they try to describe to non-prosopagnosics what they experience in everyday life. Both phenomena are characterized by the difficulty in telling people apart or recognizing previously encountered people based on their faces. But also, in both cases, there is evidence for parallels in disturbances of face processing as reviewed in the following.

Some studies used the inversion effect or the composite face effect to test face processing abilities of their participants. The inversion effect describes the effect that face recognition performance is reduced if the faces are presented upside down. The strength of this effect is significantly larger for faces than for other objects for which we are not experts. The composite face effect describes the illusion of a new identity when combining the top half of the face of one person with the bottom half face of another person. The two halves cannot be processed individually and create the face of a new, third person. The illusion disappears when the two halves are misaligned. Both effects, the face inversion and the composite face effect, are considered to be hallmarks for holistic face processing. Both disrupt the configural information leaving the featural information intact. This again is an indication of the importance of configural processing for holistic processing (Maurer et al., 2002). A study testing congenital prosopagnosic participants found no face inversion effect or composite-face effect, neither in accuracy nor in reaction times, indicating their impairment in holistic processing of faces (Avidan et al., 2011). Regarding the face inversion effect for other-race faces, two experiments testing European and Asian participants found a larger effect for same-race faces than for other-race faces in both groups of participants (Rhodes et al., 1989). When testing the composite face task with Asian and European participants, similarly, Michel and colleagues found a significantly larger composite face effect for same-race faces compared with other-race faces (Michel et al., 2006).

In a study conducted by Lobmaier and colleagues, congenital prosopagnosics were tested with scrambled faces (configural information destroyed) and blurred faces (featural information destroyed) in a delayed matching task. Prosopagnosic participants showed significantly worse performance than controls in both conditions (Lobmaier et al., 2010). Chinese and Caucasian-Australian participants tested in an old-new recognition task on blurred and scrambled Asian and Caucasian faces also showed a significantly worse performance for other-race faces than for own-race faces in both conditions (Hayward et al., 2008).

In another study, congenital prosopagnosics participants were tested on a same-different task with the so-called “Jane” set of stimuli (Le Grand et al., 2006). These stimuli faces differ either in features, configuration, or contour. Only a minority of the prosopagnosic participants performed significantly worse than controls on the faces differing in configuration or features, but most prosopagnosics performed significantly worse on faces differing in their contour. A study with Asian participants using the same “Jane” stimuli and a similarly created Asian female face set also showed only marginal effects (Mondloch et al., 2010): Chinese participants were significantly slower on other-race compared with same-race faces (analysis collapsed over all three types (features, configuration, contour), with the longest mean reaction times for the faces differing in contour) but showed no significant differences in performance for any modification (features, configuration, contour). Even though this lack of differences between groups for the “Jane” stimuli was challenged by (Yovel and Duchaine, 2006) (this will be disussed in our general discussion), we note that similar results for other-race observers and prosopagnosic observers were obtained in both studies.

There are several different causes that can reduce face recognition ability (aging, illnesses, drug consumption, etc.). However, the two face recognition disturbances under study here, CP and the ORE, seem to impair face recognition abilities in a similar way, namely by disrupting featural and configural face processing (depending on the used stimuli and task, as reviewed above) causing a lack or reduction of face expertise. Also, in both cases face recognition performance can be increased to a certain extent through training. These similarities could be a hint that the same face processing mechanisms are impaired.

To verify the hypothesis of a common underlying disturbance, it is necessary to compare in detail whether the same kind of impairments appear when looking specifically and directly at featural and configural processing. On one hand, if differences in face recognition performance appear, we can exclude a common underlying disturbance. On the other hand, if similar impairments are found, the hypothesis that the same mechanisms are disturbed is not proven, but possible. In any case, a direct comparison between CP and the ORE is a great chance to get further insights into the yet unknown mechanisms underlying face processing and face recognition.

To conduct this direct comparison we recruited three age- and gender-matched participant groups with a comparatively large sample size of 21 participants per group: German congenital prosopagnosic participants, Korean participants, and German controls. All participant groups performed the same three tests. (1) the Cambridge Face Memory Test (CFMT, Duchaine and Nakayama, 2006), an objective measure of the face recognition abilities of Caucasian faces, (2) a parametric test of the sensitivity to configural and featural information in faces; sensitivity to these two types of facial information has been shown to be reduced in congenital prosopagnosics and other-race observers in previous studies, and (3) a recognition task of faces and familiar and unfamiliar objects to test the influence of expertise on recognition performance.

As all face stimuli used in our tests were derived from Caucasian faces, we expected the Korean group to exhibit evidence of the ORE that could be compared with the performance of the prosopagnosics while the German control group would serve as a baseline. Our predictions for each test were the following: (1) For the CFMT, Koreans and prosopagnosics would have a lower score compared with German controls, due to the disadvantage in recognizing other-race faces for the Koreans and the innate face recognition impairment for the prosopagnosics. This test is a general measure of the severity of face recognition impairments and does not detect if differences in the nature of the impairments exist. (2) We expected to find a decreased sensitivity to configural and featural information for prosopagnosics and Koreans. This prediction was based on reported deficits in processing both kinds of information in prosopagnosic as well as other-race observers (Hayward et al., 2008; Lobmaier et al., 2010 respectively). If prosopagnosics and Koreans would show differences in the extraction of featural and configural information, we could exclude that common mechanisms are impaired. (3) In the object and face recognition test we expected an impaired recognition performance of the face stimuli for Koreans and prosopagnosics, again due to the disadvantage in recognizing other-race faces for the Koreans and the innate face recognition impairment for the prosopagnosics. We expected to find no differences across all participant groups in recognizing the non-expertise object stimuli. Despite a study describing that 54 congenital prosopagnosics self-reported impaired object recognition during interviews (Grüter et al., 2008), most studies explicitly testing object recognition found nearly-normal to normal object recognition abilities for prosopagnosic participants. When impairments were found, they were less pronounced than face recognition impairments (see Kress and Daum, 2003; Le Grand et al., 2006 for reviews).

## Materials and methods

### Participants

We tested three groups of participants: German congenital prosopagnosic participants (from now on referred to as “prosopagnosics”), South Korean participants (“Koreans”), and German control participants (“Germans”) with 21 participants per group. The ratio of female to male participants as well as the age of participants in each group was matched as closely as possible. Note that it was hard to recruit older male Korean participants, presumably for cultural reasons; therefore we had to resort to younger male participants in that group to have matching numbers of participants in all groups.

So far, no universally-accepted standard diagnostic tool for CP exists: while the CFMT is widely used to characterize prosopagnosic participants (e.g., Rivolta et al., 2011; Kimchi et al., 2012), other diagnostic means exist. The prosopagnosics of our study were identified by a questionnaire and interview (Stollhoff et al., 2011). Due to time constraints the Koreans and Germans did not participate in the diagnostic interview but reported to have no problems in recognizing faces of their friends and family members. To provide an objective measure of face processing abilities and to maintain comparability with other studies, we tested all participants on the CFMT and report their scores and z-scores, based on the results of the German controls, in Table 1.

**Table 1:** **Overview of the participants in the three different groups**.

	**Prosopagnosic**				**Korean**				**German**			
	**Sex**	**Age**	**CFMT**		**Sex**	**Age**	**CFMT**		**Sex**	**Age**	**CFMT**	
			**score**	**z-score**			**score**	**z-score**			**score**	**z-score**
1	f	21	38	−3.57	f	22	53	−1.05	f	23	65	0.96
2	f	22	44	−2.57	f	23	53	−1.05	f	24	69	1.63
3	f	24	37	−3.74	m	24	47	−2.06	f	24	64	0.79
4	f	27	47	−2.06	m	24	57	−0.38	f	25	57	−0.38
5	f	27	42	−2.90	m	26	51	−1.39	f	29	61	0.29
6	f	28	36	−3.91	f	28	57	−0.38	f	31	53	−1.05
7	m	33	45	−2.40	m	30	50	−1.56	m	33	59	−0.05
8	m	34	33	−4.41	m	37	53	−1.05	f	36	55	−0.72
9	f	36	38	−3.57	f	39	58	−0.22	m	36	58	−0.22
10	m	36	45	−2.40	m	41	55	−0.72	m	37	50	−1.56
11	m	37	34	−4.24	f	41	55	−0.72	f	37	64	0.79
12	f	41	34	−4.24	f	42	53	−1.05	m	39	62	0.46
13	f	46	44	−2.57	f	42	63	0.62	m	39	52	−1.22
14	f	46	39	−3.40	f	45	64	0.79	m	44	71	1,97
15	m	47	43	−2.73	f	46	44	−2.57	f	44	52	−1.22
16	m	52	40	−3.24	f	50	47	−2.06	f	46	59	−0.05
17	f	53	36	−3.91	f	51	63	0,62	f	47	54	−0.89
18	f	54	46	−2.23	f	55	54	−0.89	f	49	58	−0.22
19	m	57	37	−3.74	f	55	38	−3.57	m	54	68	1.46
20	m	59	38	−3.57	f	57	50	−1.56	f	58	54	−0.89
21	f	64	38	−3.57	f	58	50	−1.56	m	60	60	0.12
Mean scores			39.71	−3.28			53.10	−1.04			59.29	0.00
♂	8				6				8			
Mean age		40.2				39.8				38.8		

*Depicted are their sex (f, female; m, male), age in years, and their scores in the CFMT as well as the according z-scores, based on the results of the German controls*.

All participants provided informed consent. All participants have normal or corrected-to-normal visual acuity.

#### German congenital prosopagnosic participants

The prosopagnosics were diagnosed by the Institute of Human Genetics, Universitäts-klinikum Münster, based on a screening questionnaire and an diagnostic semi-structured interview (Stollhoff et al., 2011). All prosopagnosics were tested at the Max Planck Institute for Biological Cybernetics in Tübingen, Germany and compensated with 8 Euro per hour plus travel expenses.

#### Korean participants

The Korean participants were compensated with 30,000 Won (approximately 20 Euro) for the whole experiment. All participants of this group were tested at Korea University in Seoul, South Korea. The Koreans did not perform a diagnostic interview but were asked if they had noticeable problems recognizing faces of friends and family members. None of the participants reported face recognition impairments.

#### German control participants

The German control participants were compensated with 8 Euro per hour. All participants of this group were tested at the Max Planck Institute for Biological Cybernetics in Tübingen, Germany. The Germans did not perform a diagnostic interview but were asked if they had noticeable problems recognizing faces of friends and family members. None of the participants reported face recognition impairments.

### Analysis

Many studies found faster reaction times for Asian compared with Caucasian participants regardless of the task (Rushton and Jensen, 2005). We made similar observations in our study and hence we do not compare reaction times between our Asian and Caucasian participants, as any comparison would not give interpretable results. Nevertheless, we compared reaction times for prosopagnosics and Germans for the object recognition task, as participants in both groups share the same ethnicity.

All analyses were conducted with Matlab2011b (Natick, MA) and IBM SPSS Statistics Version 20 (Armonk, NY). The dependent variables analyzed in each test are described in the respective sections.

We report effect sizes as partial eta square (η^2^_*p*_). For One-Way ANOVAs partial eta square and eta square (η^2^) are the same. For our Two-Way ANOVAs partial eta square differs from eta square, therefore we give both values.

### Apparatus

All participants were tested individually. For prosopagnosics and Germans the experiments were run on a desktop PC with 24″ screen, Koreans performed the tests on a MacBook Pro with a 17″ screen. The CFMT is Java-script based; Matlab and Psychtoolbox were used to run the other experiments. Participants were seated at a viewing distance of approximately 60 cm from the screen.

### Procedure

The procedure was approved by the local IRB. All participants completed three tests: (1) the CFMT, (2) a rating task of the similarity of faces differing in features or configuration, (3) an object recognition task. All tests were conducted in the same order to obtain comparable results for each participant. Participants could take self-paced breaks between experiments.

## Test battery

### Cambridge face memory test

#### Motivation

The CFMT was created and provided by Bradley Duchaine and Ken Nakayama (Duchaine and Nakayama, 2006). This test assesses recognition abilities using unfamiliar faces in a 3-alternative-forced-choice task. It has been widely used in recent years in studies of CP and of the ORE. Therefore, we used it here as an objective measure of face recognition abilities.

#### Stimuli

As this test has been described in detail in the original study, only a short description is given here. Pictures of the faces of young male Caucasians shown under three different viewpoints and under different lighting and noise conditions were used in recognition tests of increasing difficulty. For a complete description of the test see the original study (Duchaine and Nakayama, 2006).

#### Task

First the participants were familiarized with six target faces which they then had to recognize among distractors in a 3-alternative-forced-choice task with tests of increasing difficulty. No feedback was given. The test can be run in an upright and inverted condition. We only used the upright condition.

#### Results

**Figure 1:**
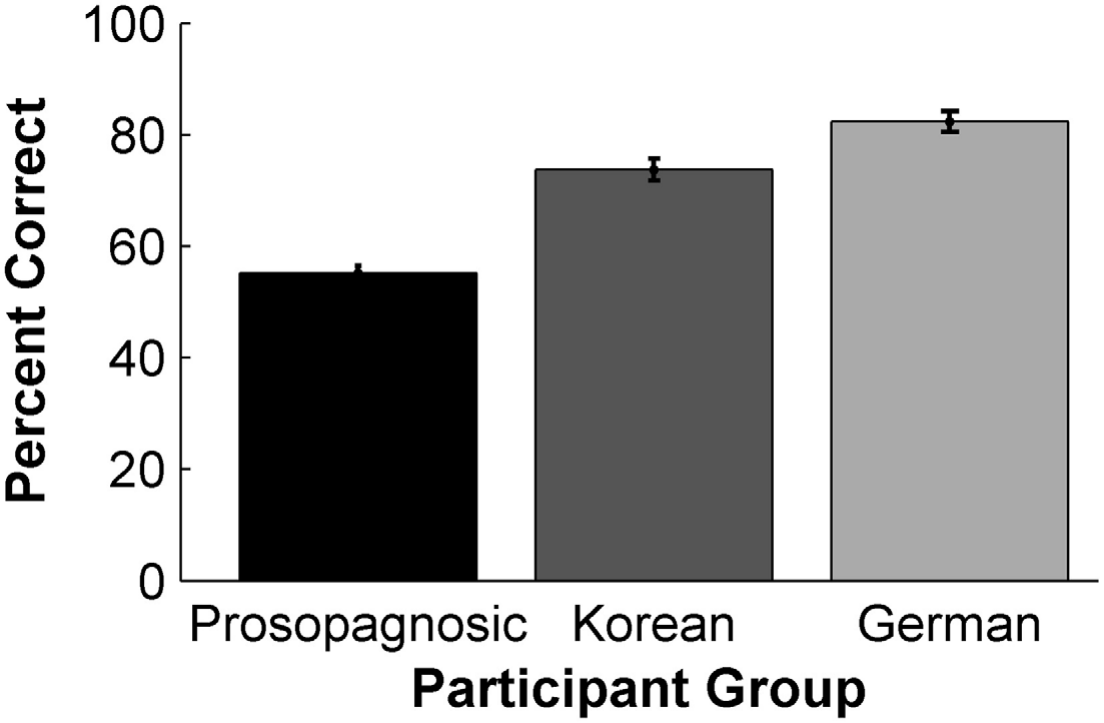
**Performance of the 3 participant groups in the CFMT**. Data are displayed as mean percentage correct responses. Error bars: SEM.

The percent correct recognition of participants was calculated and the mean and standard error of the three participants groups are depicted in Figure 1.

Germans (mean percent correct = 82.3%, *SD* = 8.3) performed significantly better than Koreans (mean = 73.7%, *SD* = 8.8), who performed significantly better than prosopagnosics (mean = 55.2%, *SD* = 5.9) [One-Way ANOVA: *F*_(2, 62)_ = 67.34, *p* < 0.001, η^2^_*p*_ = 0.69, with Tukey HSD *post-hoc* tests: all comparisons *p* ≤ 0.002].

#### Discussion

As predicted, the Koreans and prosopagnosics performed significantly worse than the Germans. Furthermore, the prosopagnosics performed significantly worse than the Koreans. The significant difference in performance for the Germans and Koreans shows an own-race advantage for the Germans. We assume that reduced performance of the Koreans is due to the ORE; however, as we did not perform the reverse test with Asian faces, we cannot completely exclude an alternative cause for this difference between participant groups. We suggest that this is very unlikely, because the CFMT and its Chinese version (comprising Chinese faces depicted in a similar way and format as the faces in the CFMT; only published after our data acquisition) were already successfully used to measure the ORE in a complete cross-over design in Caucasian and Asian participants (McKone et al., 2012).

From our finding that Koreans show a significantly better recognition performance than prosopagnosics we cannot exclude that the same mechanisms for processing Caucasian faces are affected in these groups. But we can infer that CP has a stronger impact on face recognition abilities compared with the ORE.

### Similarity rating of faces differing in features or configuration

#### Motivation

This test was conducted to measure in what way and to what extent the retrieval of featural and configural information is disturbed in other-race observers and prosopagnosics. Based on this pattern we want to infer if we can exclude that the same mechanisms for processing Caucasian faces are affected in CP and the ORE. As discussed in the introduction, previous studies found disturbances in holistic processing (e.g., Avidan et al., 2011 for CP; Rhodes et al., 1989; Michel et al., 2006 for the ORE), and disruptions of configural and featural processing (e.g., Lobmaier et al., 2010 for CP; Hayward et al., 2008 for the ORE). However, other studies using different tasks and stimuli found only minor or no impairments in configural and featural processing (e.g., Le Grand et al., 2006 for CP; Mondloch et al., 2010 for the ORE). The pattern of findings obtained so far was too inconsistent and not detailed enough to draw conclusions regarding our research question. To resolve this controversy and to obtain usable data, we assessed the fine-grained sensitivity to featural and configural facial information and compared the effects of CP and the ORE.

#### Stimulus creation

We generated eight natural-looking face sets with gradual small-step changes in features and configuration to determine the grade of sensitivity to featural and configural facial information, without resorting to unnatural modifications (like blurring or scrambling). The faces in each of our stimulus sets differ only in internal features and their configuration. Skin texture and outer face shape were held constant to allow testing purely for sensitivity to internal features and configuration. The face stimuli contain no extra-facial cues (no hair, makeup, clothing, or jewelry).

**Figure 2:**
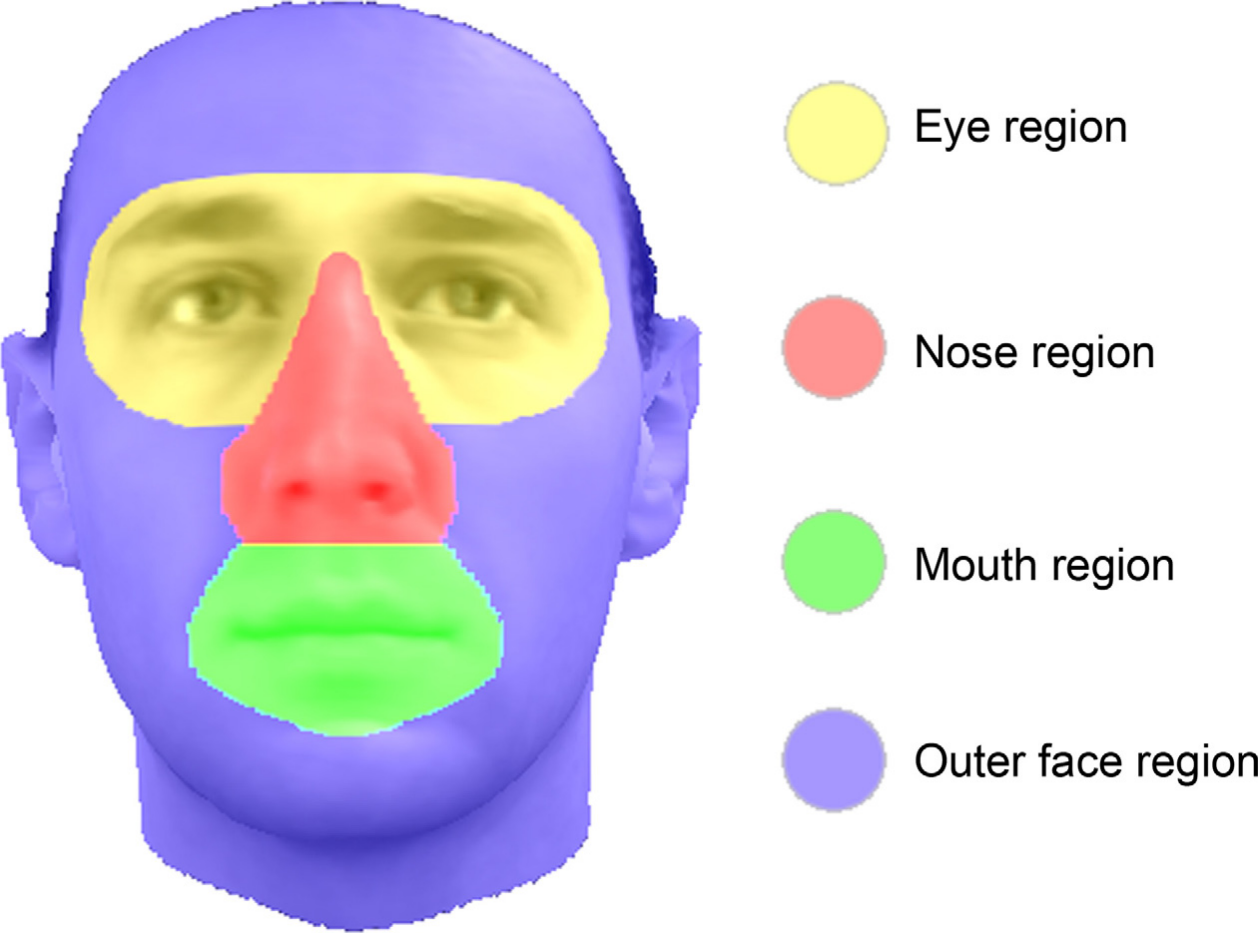
**Illustration of the editable regions of the 3D faces of our in-house face database (Troje and Bülthoff, 1996)**.

The stimuli were created using faces from our in-house 3D face database (Troje and Bülthoff, 1996). The faces are 3D laser scans of the faces of real persons. A morphable model allows to isolate and exchange the four main face regions between any faces of the database (Vetter and Blanz, 1999). Those four regions are: both eyes (including eyebrows), the nose, the mouth, and the outer face shape (Figure 2). For these regions, the texture (i.e., “skin”) and / or the shape can be morphed as well as exchanged between all faces. Additionally the regions can be shifted within each face (e.g., moving the eyes up or apart of each other).

We chose pairs of faces from the database such that the faces in each pair differed largely from each other in both configuration and features. Previous studies that have used faces differing in either features or configuration have shown that participants are more sensitive to featural than to configural changes (Freire et al., 2000; Goffaux et al., 2005; Maurer et al., 2007; Rotshtein et al., 2007). For this reason we further increased the configural differences of the face pairs by shifting the features slightly (e.g., we moved the eyes closer together in the face which had more closely spaced eyes, and moved the eyes further apart in the other face of the pair). This was done for best conditions to measure configural sensitivity, as this is one main focus of our study, while remaining within natural limits. That the faces are still perceived as natural was tested in a pilot study described further below.

**Figure 3:**
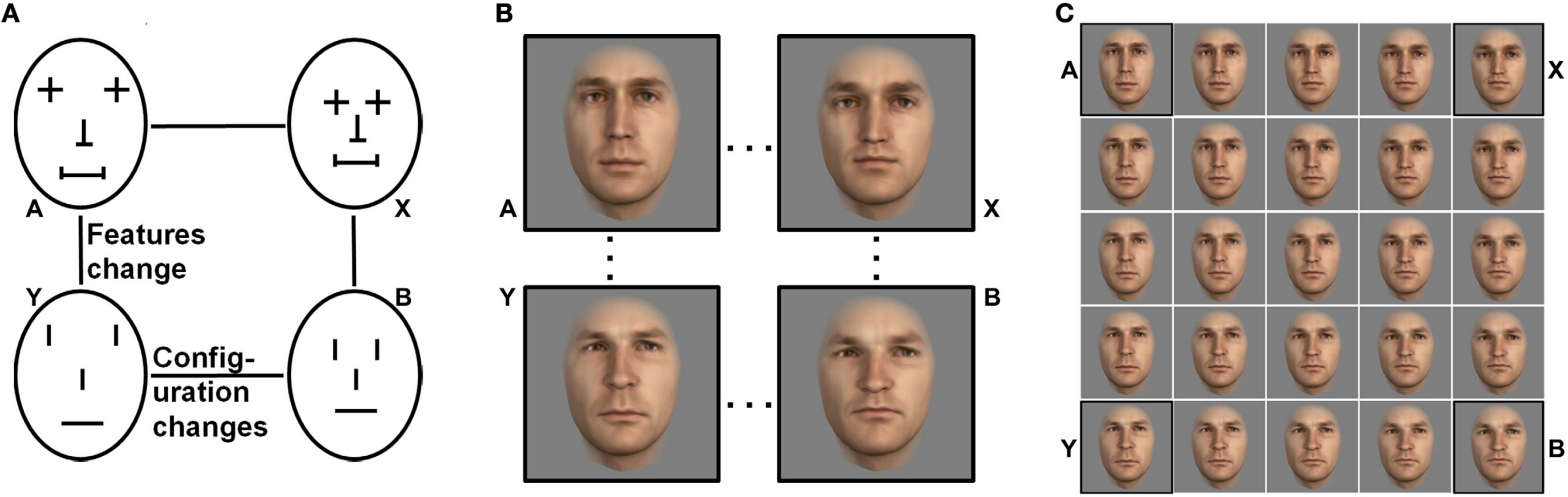
**(A)** Schematic four faces which either differ in features (horizontal) or configuration (vertical). **(B)** The same design is applied to real faces of our face database. **(C)** Morphing between the four faces in **(B)** gives a set. Morphing steps between each row and column are equally spaced with 25%.

The outer face shape and skin texture of the modified faces were averaged within each pair and applied to both modified faces to create two faces A and B (Figure 3B). A and B exhibit different features and inner configuration but identical averaged outer face shape and skin texture. Based on the faces A and B we then generated two more faces by creating a face X with features of face A and the configuration of face B (i.e., the features of face A were moved to the feature locations of face B) and vice versa for face Y (see scheme in Figure 3A; see actual face stimuli in Figure 3B). By morphing between these four faces in 25% increments we generated a whole set of faces parametrically differing from each other in features (Figure 3C, horizontal axes) or configuration (Figure 3C, vertical axes). We created eight different sets in the same way as the one depicted in Figure 3C, one for each of eight pairs of original faces of our database (note: each original face was used only in one set).

To ensure that the faces we created appeared just as natural as the original faces, we ran a pilot study in which participants rated the naturalness of the modified and original faces without any knowledge about the facial modifications. The modified faces we used for our study showed no significant difference in perceived naturalness compared with the original scanned faces of real people (Esins et al., 2011).

**Figure 4:**
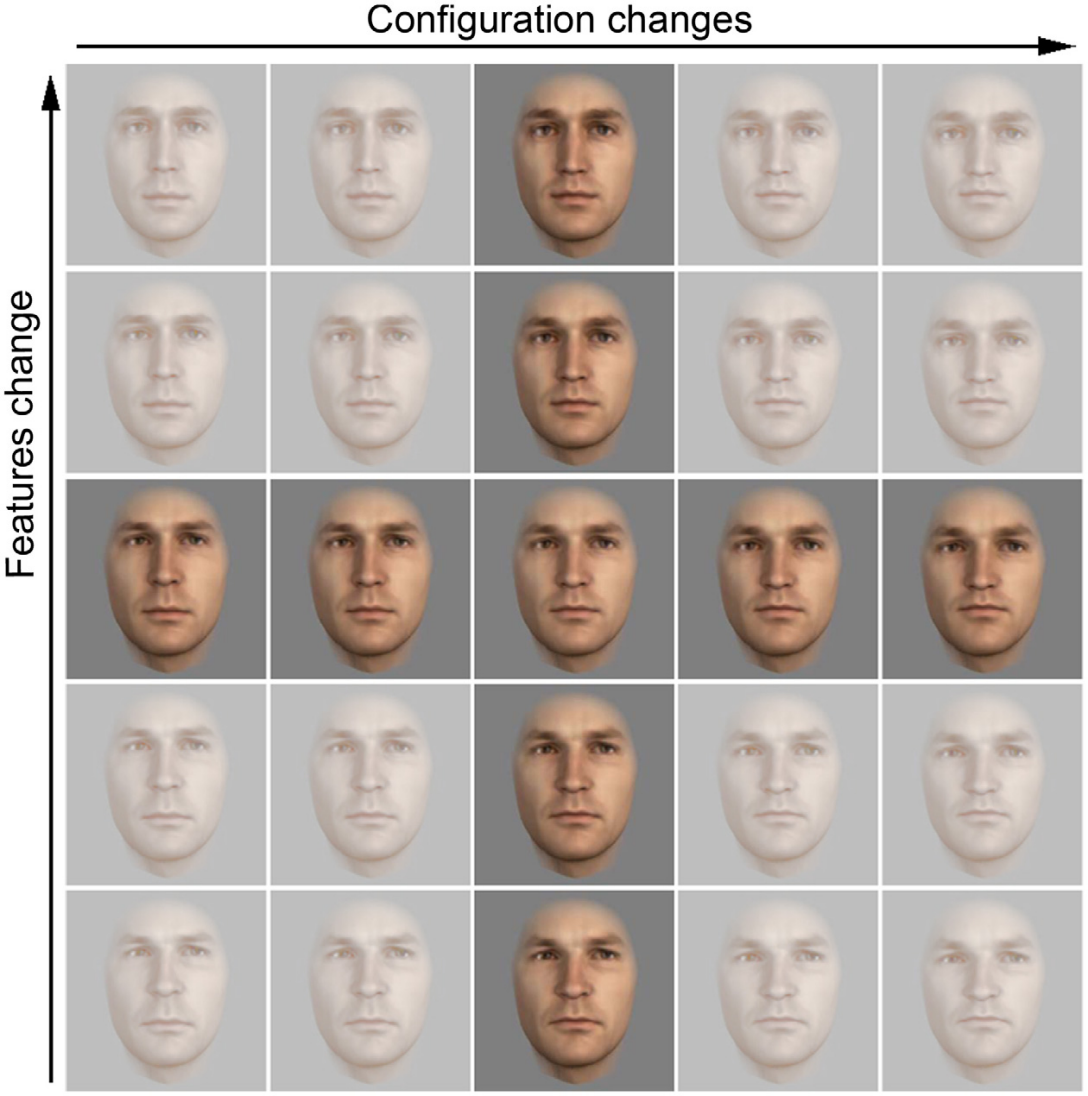
**One of the eight sets of face stimuli used in the similarity rating experiment**. Only faces of the central horizontal and vertical bars were used for the experiments. The endpoint faces were used to calculate mean pixelwise image differences between the stimuli.

Further, to verify that featural and configural modifications introduced similar amounts of changes in the pictures, we calculated the mean pixelwise image differences between the stimuli with the greatest configural and featural parametrical differences per set. We took the two end point faces of the vertical bar (see Figure 4) and calculated their Euclidean distance for each pixel and did the same for the two end point faces of the horizontal bar. Then we calculated the average pixel distance for the two comparisons^1^. With this method we obtained mean Euclidean pixel distances for configural and featural changes, for each of the eight created sets. A Wilcoxon signed rank test run on all eight mean distances for the featural changes vs. the eight configural change distances was not significant (*p* = 0.31), supporting the idea that featural and configural face modifications introduced similar amounts of computational change in the pictures.

#### Task

Participants had to rate the pair-wise similarity of faces originating from the same set. Due to time limitations we used only nine test faces per set: the ones located on the central horizontal bar (differing in features) and the central vertical bar (differing in configuration) of each set (see Figure 4). Each face was compared with the eight other faces on the central bars of the same set and with itself. Trials in which faces differed in both, features and configuration, were considered filler trials to avoid participants realizing the nature of the stimuli and were omitted from the analysis. Therefore, in sum, for each of the eight sets, we analyzed 29 pair-wise similarity ratings: nine identical face comparisons (100% parametrical similarity), eight face comparisons with 75% parametrical similarity (two faces next to each other in the set), six face comparisons with 50% parametrical similarity, four face comparisons with 25% parametrical similarity, and two face comparisons with 0% parametrical similarity (comparison of the extreme faces of the same bar). So in total there were 232 comparisons during this experiment. The order of comparisons was randomized within and across sets for each participant.

**Figure 5:**
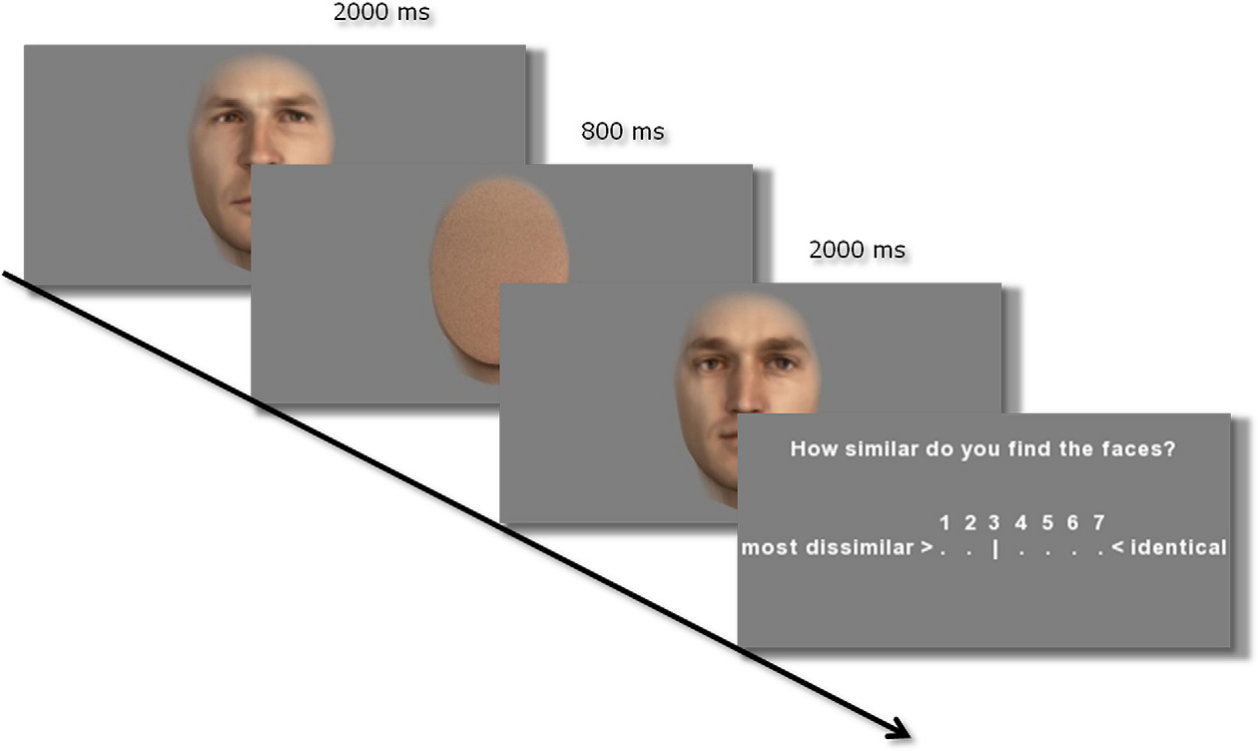
**Example of one trial of the similarity rating task**. Both faces in a trial always belong to the same set.

Participants had to rate the perceived similarity on a Likert scale from 1 (little similarity) to 7 (high similarity/identical) and were told to use the whole range of ratings over the whole experiment. The participants saw the first face for 2000 ms, then a pixelated face mask for 800 ms, and then the second face for another 2000 ms. Subsequently, the Likert scale appeared on the screen: here participants marked their rating by moving a slider via the arrow keys on the keyboard (Figure 5). The start position of the slider was randomized. There was no time restriction for entering the answer, however, participants were told to rate the similarity without too long considerations. After every 20 comparisons there was a self-paced pause.

The face and mask stimuli had a size of approximately 5.7° horizontal and 8.6° vertical visual angle. To prevent pixel matching, the faces were presented at different random positions on the screen within a viewing angle of about 7.6° horizontally and 10.5° vertically.

#### Analysis

**Figure 6:**
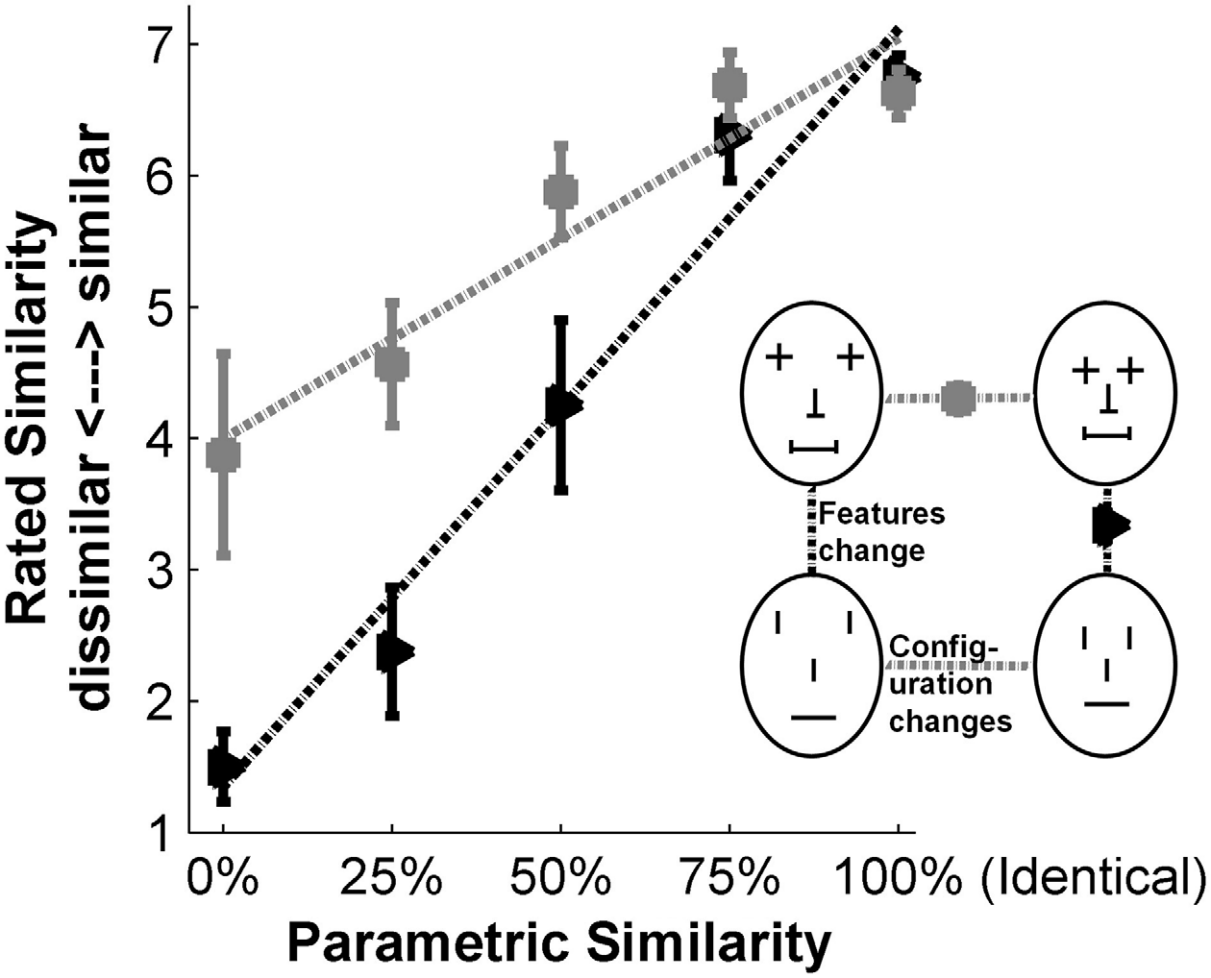
**Exemplar results of one German participant of the similarity ratings**. For each of the five similarity levels, the average ratings across all face comparisons of all sets were calculated. The sensitivity ratings for changes in features (black triangles) and configuration (gray squares) are shown separately. The error bars depict standard error. A linear regression (*y* = β*x* + ε) was fitted to both curves individually (dotted black and dotted gray, respectively). The slopes (β) serve as measure of the sensitivity to features and configuration.

For every participant we calculated the mean similarity ratings across all eight sets at each of the five levels of parametric similarity (100, 75, 50, 25, 0%). Example data of one German participant is given in Figure 6. The black triangles show the average rating of face pairs of all sets differing in features, sorted by the different parametrical similarities. The gray squares show the same for configural changes. As expected, Germans gave similarity ratings close to 7 (high similarity) for very similar faces.

**Figure 7:**
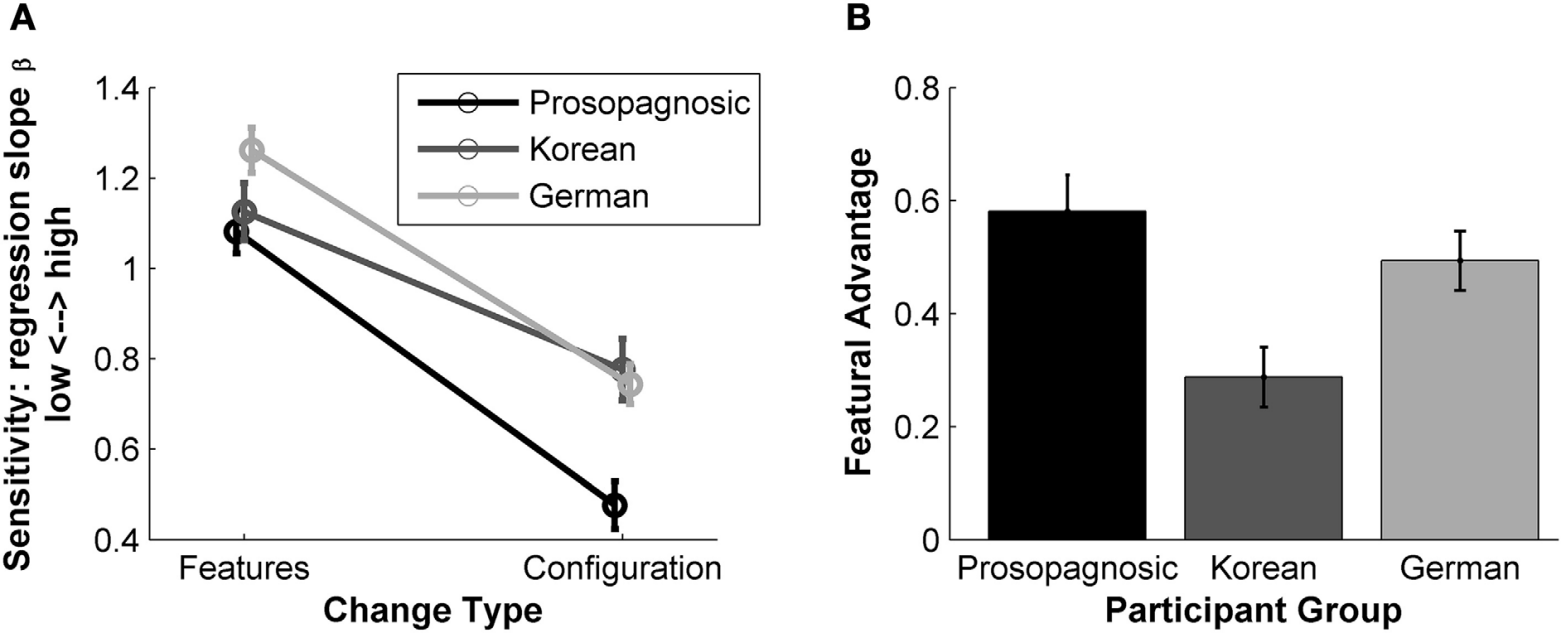
**Results of the similarity rating experiment**. **(A)** Mean values of slopes (β) for the “feature” and “configuration” regression lines for each group Error bars: SEM. **(B)** “featural advantage”: mean difference between configural and featural regression slopes (β) calculated for each participant. Error bars: SEM.

A linear regression (*y* = β*x* + ε) was fitted to these mean similarity ratings (dotted black and gray lines in Figure 6). The steepness of the slopes (β) was then used as a measure of sensitivity: steeper slopes indicate more strongly perceived configural or featural changes. For every participant we calculated one regression slope for their featural and one for their configural ratings. The mean and the standard error of the sensitivity β per participant group are illustrated in Figure 7A.

To compare performance data, we took a closer look at the pattern of sensitivity to features and configuration: For each individual participant, we subtracted their configural sensitivity from their featural sensitivity. We refer to this difference as ‘featural advantage’. The illustration in Figure 7B shows the mean of the calculated differences, i.e., the mean of the featural advantage for each group.

#### Results

A 2 × 3 ANOVA on the regression slopes β as a measure of sensitivity showed that the main effect of change type (configural, featural) was significant [*F*_(1, 60)_ = 233.7, *p* < 0.001, η^2^ = 0.46, η^2^_*p*_ = 0.796]. All participants showed a greater sensitivity to changes in features than to changes in configurations. The main effect of participant group (prosopagnosics, Koreans, Germans) was also significant [*F*_(2, 60)_ = 6.46, *p* = 0.003, η^2^ = 0.07, η^2^_*p*_ = 0.18]. The interaction between change type and participant group was significant, too [*F*_(2, 60)_ = 5.48, *p* = 0.007, η^2^ = 0.02, η^2^_*p*_ = 0.15].

Analysis of simple effects for both change types (configural, featural) was carried out: The group differences of sensitivity to features approaches significance [One-Way ANOVA *F*_(2, 62)_ = 3.12, *p* = 0.0515, η^2^_*p*_ = 0.09], which was mainly driven by the difference between prosopagnosic and Germans (Tukey HSD *post-hoc* test, *p* = 0.051, both other differences *p* > 0.17). For configural changes there were significant group differences in sensitivity [One-Way ANOVA *F*_(2, 62)_ = 9.11, *p* < 0.001, η^2^_*p*_ = 0.23] with prosopagnosics performing significantly differently from Koreans and Germans (Tukey HSD *post-hoc* test, *p* = 0.001 and *p* = 0.003, respectively. Tukey HSD *post-hoc* test for Koreans vs. Germans *p* = 0.91).

For analysis of the featural advantage (Figure 7B) we conducted a One-Way ANOVA to further examine the significant interaction of the main effects (participant group vs. change type). The ANOVA showed significant differences between the three groups [*F*_(2, 62)_ = 5.48, *p* = 0.007, η^2^_*p*_ = 0.15], which are the same values as for the interaction in the 2 × 3 ANOVA, as expected. The Tukey HSD *post-hoc* tests revealed significant differences in the featural advantage between Koreans and prosopagnosics (*p* = 0.005), a difference approaching significance for the Koreans vs. the Germans (*p* = 0.091) and no difference for prosopagnosics vs. Germans (*p* = 0.51).

#### Discussion

There is a clear difference in sensitivity to features and configuration of our stimuli faces between Koreans and prosopagnosics: while both groups show about the same sensitivity to featural changes, we found that prosopagnosics have a significantly reduced sensitivity to configuration compared with Koreans (and Germans). Also the featural advantage was significantly smaller for Koreans than for the prosopagnosics. These differences in absolute sensitivity to configural and featural changes, and also the differences in featural advantage, suggest that Korean and prosopagnosic participants do not perceive our Caucasian face stimuli in the same way. Because CP and the ORE show parallels in disrupting featural and configural face processing, we hypothesized that the same mechanisms are disturbed in both cases. This would result in a similarly reduced sensitivity to features and configuration for participants affected by CP or the ORE. But as Korean and prosopagnosic participants show a different pattern of disturbance of their sensitivity, we can reject this hypothesis and conclude that different underlying mechanisms are affected.

Our similarity rating task also allowed to obtain a more detailed picture of the sensitivities to featural and configural information in CP and the ORE. For the prosopagnosics compared with the Germans, the difference between both groups approached significance for sensitivity to features and reached significance for sensitivity to configuration (Figure 7A). Our results show a marginally significant difference for prosopagnosics and Germans in featural sensitivity (*p* = 0.051). These results bridge the gap between two studies reporting conflicting results using the so-called “Jane” stimuli (Le Grand et al., 2006) and “Alfred” stimuli (Yovel and Kanwisher, 2004; Yovel and Duchaine, 2006), which, like our stimuli, also differ in features and configuration (and contour for the “Jane” stimuli). Only a minority of the prosopagnosic participants performed significantly worse than controls on the “Jane” stimuli differing in features and configuration (Le Grand et al., 2006). Based on the data by Le Grand and colleagues given in Table 4 of that study, comparing prosopagnosics and controls, one can estimate that there was a significant performance difference for the configural but not for the featural modifications. Yovel and colleagues also used the “Jane” stimuli with prosopagnosics and controls and confirmed the significant performance difference between groups for configural modifications and non-significant difference for featural modifications (Yovel and Duchaine, 2006). However, they challenged the “Jane” stimuli for including obvious brightness differences (due to makeup) for the featural modifications. For their own “Alfred” stimuli they found significantly reduced sensitivity to featural and configural modifications for prosopagnosic participants (Yovel and Duchaine, 2006; Duchaine et al., 2007). In turn, their “Alfred” stimuli were challenged for configural modifications going beyond natural limits (as discussed in Maurer et al., 2007). Our newly created stimulus set contains no extra-facial cues (no hair, makeup, glasses, or beard) and exhibits configural changes which have been tested to be within natural limits. With these well controlled stimuli our results suggest that for prosopagnosic participants, the retrieval of the configural information of a face is indeed impaired compared with the Germans. For the sensitivity to features, our results lie between the non-significant results obtained with the “Jane” stimuli and the significant results obtained with “Alfred” faces. Therefore, we conclude that the retrieval of featural information might be impaired for prosopagnosics, although to a lesser degree than the retrieval of configural information.

We found no significant difference in sensitivity to featural or configural information between the Korean and German groups. Our result are in concordance with a previous study, also using the “Jane” stimuli, that found no differences between Caucasian and Asian participants (Mondloch et al., 2010). In contrast, other studies found an own-race advantage for both configuration and feature changes (Rhodes et al., 2006; Hayward et al., 2008). However, we note that the stimuli used in those latter studies involved different kinds of changes than those used in our present study (features and configuration were changed by blurring and scrambling (Hayward et al., 2008) or features were changed through changes in color (Rhodes et al., 2006), which opens the possibility that the ORE impacts differently on the perception of these different kinds of stimulus modifications. Nevertheless, as our stimuli contain more natural and ecological modifications of faces, we believe that our results better reflect participants' face perception. Even though we found no significant differences in sensitivity to featural or configural information between Germans and Koreans, we found that the featural advantage shows a trend to be larger for the Germans compared with the Koreans. Although this difference only approaches significance, we present two explanations for this pattern. The first explanation is that due to the ORE, the sensitivity pattern is altered for our Korean participants. The ORE could reflect Koreans' lower expertise with other-race facial features whereas their configural processing stays unaffected when viewing other-race faces. The second explanation is that the effect is due to cultural differences. Studies have shown that Western Caucasian and Eastern Asian participants focus at different areas of faces and have dissimilar patterns of fixation when looking at faces (Blais et al., 2008). It might be that German and Korean participants employ different strategies when comparing faces in our task, which could have caused the effects we found. In accordance with this hypothesis, a study using Navon figures reported that Eastern Asian participants focus more on global configuration compared with Western Caucasian participants (McKone et al., 2010). By analogy, a greater focus on configurations in faces could explain the reduced featural advantage we observed in the Korean group.

Furthermore, our results show that all groups, regardless of their race and face recognition abilities, were more sensitive to differences in the featural than in the configural dimension of our stimulus set (Figure 7A). The presence of a featural advantage is in accordance with findings of previous studies using faces modified within natural limits in their configuration and features, where participants showed a higher sensitivity for featural changes as well (Freire et al., 2000; Goffaux et al., 2005; Maurer et al., 2007; Rotshtein et al., 2007). Even though for the “Alfred” stimuli similar sensitivities to featural and configural modifications were found by Yovel and Kanwisher (2004), their result should be regarded with caution in view of the unnatural configural modifications of their face stimuli (as discussed in Maurer et al., 2007). In contrast, we took care that our face stimuli were always natural looking and pixelwise analyses of our stimuli, as described earlier, have revealed no differences in induced image changes in the featural and configural dimensions. In other words, our stimuli exhibit the same pixelwise variation for the featural and configural changes. The fact that the observers nevertheless show a featural advantage suggests that humans are more sensitive to featural information, and/or perceive these changes to be more profound than changes in configuration. Another possible explanation is that it is more difficult to compare faces differing in configuration than to compare faces differing in features. Additionally, differences between two naturally-occurring faces are more likely to be featural than configural. Therefore, the human face discrimination system might have developed to be better at detecting featural than featural differences between faces.

### Object recognition

#### Motivation

In this test we measured the influence of expertise on recognition performance. To this end, we compared recognition performance for objects for which one group has expertise (Caucasian faces) to recognition performance for objects for which no group has expertise (seashells and blue objects).

#### Stimulus creation

**Figure 8:**
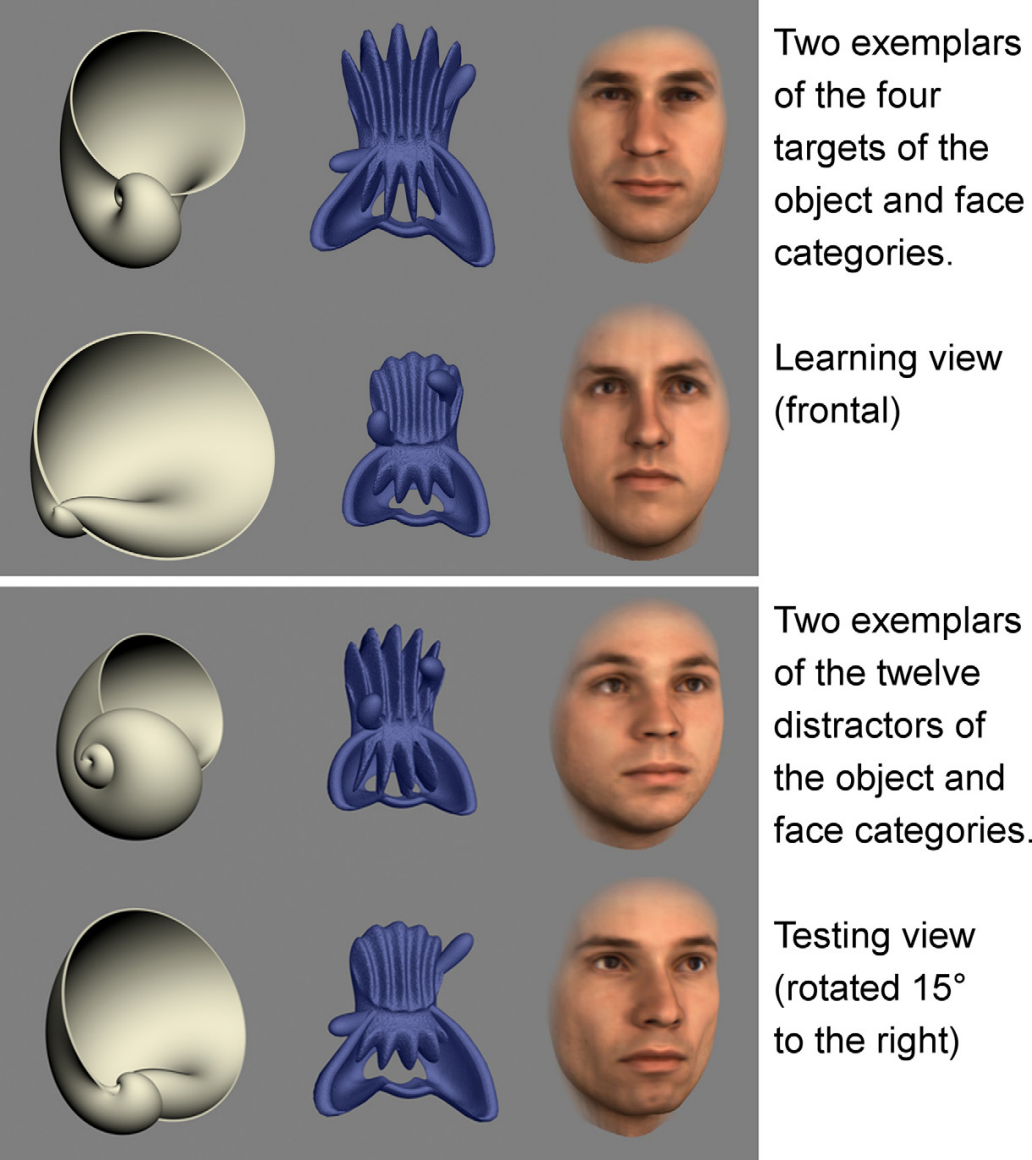
**Exemplars of the stimuli used in the object recognition experiment**.

Three categories of stimuli were used: computer renditions of natural objects (seashells), artificial novel objects (blue objects, dissimilar to any known shapes) and faces. See Figure 8 for examples of these three categories of objects. All objects and faces where full 3D models, allowing to train and test participants on different viewpoints (see below). For each category we created four targets and twelve distractors.

Sixteen synthetic seashells were taken from a previously created stimulus set (Gaißert et al., 2010). The shells were created using a mathematical model (Fowler et al., 1992) implemented in the software ShellyLib (www.shelly.de). Attention was paid to sample stimuli spread evenly over the parametrically defined stimulus set space (see Gaißert et al., 2010 for details).

The blue objects were created with 3D Studio Max by Christoph D. Dahl (unpublished work) and were novel to all participants. Differences between these objects are less obvious for a human observer, making recognition more difficult.

For the face stimuli, 16 male Caucasian faces were selected from the MPI 3D face database (Troje and Bülthoff, 1996). The 16 faces where chosen to have as little salient distinctive features as possible (all were clean shaven, had the same gaze direction; showed no blemishes or moles, etc).

None of the stimuli had been seen before by our participants. We created two sets of images for each stimulus category: frontal views for the learning phase, and stimuli rotated by 15 degrees to the right around the vertical axis (yaw) for the testing phase. The change between learning and testing was designed to prevent pixel matching of the stimuli.

All stimuli were shown at a viewing angle of approximately 9.5° horizontally and vertically.

#### Task

There was one block of trials per stimulus category, with the same procedure in all three blocks, as follows: During the learning phase, participants had to memorize four target exemplars depicted in frontal view. First, all four targets were shown together on the screen, then each of the four targets was shown one after the other, and finally all target exemplars were presented together again. Participants could control when to switch to the next screen via a button press. They were aware that if they switched to the next view they could not return to the previous one. No time restriction was applied. During testing, participants saw the images depicting the targets and distractors of the same category under a new orientation and performed an old-new-decision task by pressing buttons on a standard computer keyboard (old = left hand button press; new = right hand button press). Stimuli were presented for a duration of 2000 ms or until key press, whichever came first. The next image appeared as soon as an answer was entered.

Targets and distractors were presented in pseudo-randomized order: The testing was divided into three runs. Four targets and four distractors per category were shown in each run. While the targets were the same in each run, four new distractors were presented, such that all four targets were seen three times and each of the 12 distractors was seen only once. The order of the stimulus blocks (shells, faces then blue objects) was fixed to induce similar effects of tiredness in all participants. Participants took short self-paced breaks between blocks.

We kept the number of targets and distractors low, as performing tests with faces can be demotivating for prosopagnosics. We used the same number of stimuli in all stimulus categories to ensure comparability. The high similarity between the non-face objects was designed to avoid ceiling performance despite the low number of stimuli and to mimic the homogeneity of the face stimuli.

#### Analysis

The results were analyzed based on the dependent measure *d*′. The term *d*′ refers to signal-detection theory measures (Macmillan and Creelman, 2005) and is an index of subjects' ability to discriminate between signal (target stimuli) and noise (distractors). The maximum possible *d*′ value in this experiment is 3.46 (this depends on the number of trials). A *d*′ of zero indicates chance discrimination performance, higher values indicate increasing ability to tell targets and distractors apart.

#### Results

**Figure 9:**
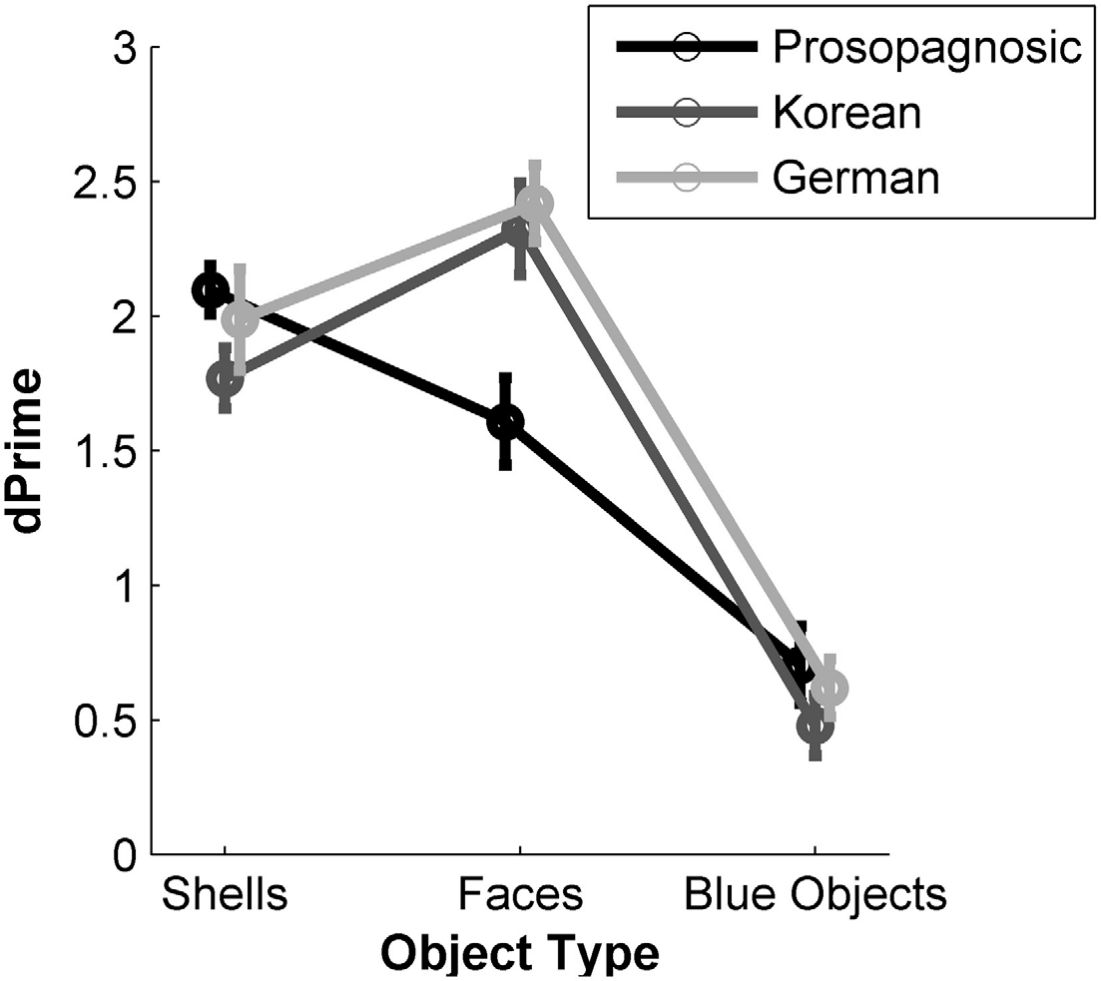
**Performance of the three participant groups in the object recognition task**. Data are shown as mean *d*′ values. Error bars: SEM.

For a summary analysis of the general influence of object category (faces, shells, blue objects) and participant group (prosopagnosics, Koreans, Germans) we ran a 3 × 3 ANOVA on the *d*′ values. The main effect of participant group was not significant [*F*_(2, 60)_ = 1.22, *p* = 0.303, η^2^ = 0.009, η^2^_*p*_ = 0.04] but the main effect of object category was [*F*_(2, 60)_ = 145.54, *p* < 0.001, η^2^ = 0.52, η^2^_*p*_ = 0.71], as well as the interaction between participant group and object category [*F*_(4, 120)_ = 7.14, *p* < 0.001, η^2^ = 0.05, η^2^_*p*_ = 0.19]. Figure 9 depicts the performance of all groups graphically. The Germans and the Koreans were better at recognizing faces than shells and worst for recognizing the blue objects. This order differs for the prosopagnosics who were best at recognizing shells, faces and blue objects in that order.

A One-Way ANOVA on the *d*′ values for each object category across participant groups revealed significant differences for the face stimuli: *F*_(2, 62)_ = 8.14, *p* = 0.001, η^2^_*p*_ = 0.21. A *post-hoc* analysis showed that prosopagnosics' performance was significantly different from the other two groups (Games Howel test, *p* ≤ 0.01 for prosopagnosics vs. Koreans and prosopagnosics vs. Germans). The other One-Way ANOVAs and *post-hoc* tests on the level of shells and blue objects, respectively, were not significant (all *p*s > 0.2).

We also compared reaction times of Germans and prosopagnosics for the non-face object categories (shells, blue objects) with the Wilcoxon Rank sum test. We found no significant differences (*p* = 0.13 for shells, *p* = 0.31 for blue objects).

#### Discussion

As expected, no significant differences between groups were found for shells and blue objects. This can be explained by the fact that all participants, equally, were non-experts for these objects. Performance differed only for faces. We found that prosopagnosics, as non-experts for faces, performed less well on face recognition than the other two groups. Interestingly, the Koreans, also non-experts for our Caucasian stimuli, did not exhibit a lower recognition performance than Germans. An obvious reason for the absence of the ORE is the small amount of targets to be memorized for this test. It is thus likely that the task was too easy for all non-prosopagnosic participants. For the prosopagnosics, our results show that the task is difficult even with this small amount of target faces. This confirms the results we observed in the CFMT, namely that CP has a stronger impact on face recognition abilities compared with the ORE.

We compared recognition performance for faces not only with one type of objects but with easy and difficult object categories, which reduces the risk of ceiling or flooring effects. Germans and Koreans recognized the non-face objects less easily than the faces, probably because, even for Koreans, their expertise for faces is better than their expertise for the visually similar non-face objects. For prosopagnosics the accuracy performance for faces lay between their performance for easy and difficult object categories. This indicates that the stimuli were not too easy to recognize.

Our findings confirm previous results indicating that, although some prosopagnosics might show object recognition deficits, those impairments are less severe than their face recognition deficits (Kress and Daum, 2003; Le Grand et al., 2006). But a further aspect of object recognition expertise worth exploring is reaction times. Behrmann and colleagues found that object recognition deficit of their five prosopagnosic participants does not show in accuracy performance, but in reaction time (Behrmann et al., 2005); and in a study by Duchaine and Nakayama (2005), many prosopagnosic participants exhibited longer reaction times rather than lower recognition accuracy compared with control participants: four of their seven prosopagnosic participants had a reaction time slower by more than 2 SD compared with the mean reaction time of their controls in most tasks. We did not find slower reaction times for non-face object recognition for prosopagnosics compared with Germans. These results thus exclude a general recognition deficit in our prosopagnosics.

## Correlations between tests

Given that we ran several face processing experiments with different tasks testing for different aspects of recognition, we also examined the degree of correlation between test performances. For this we calculated Pearson's correlations between task performances across participants of all groups (Table 2).

**Table 2:**
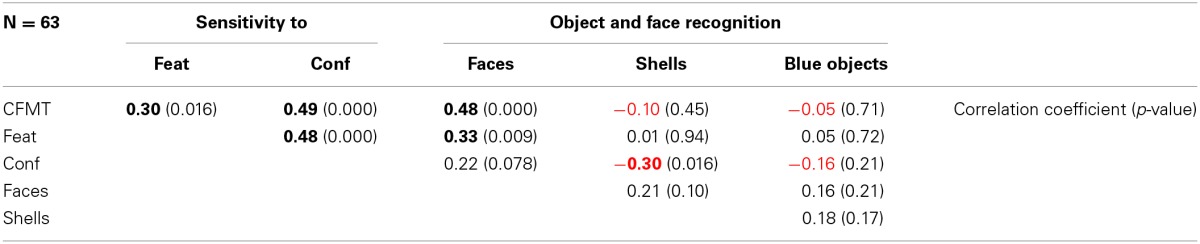
**Pairwise correlations between test scores of all participants combined**.

Depicted are the correlation coefficient, and in parentheses the p-value of the coefficient. Negative correlations are marked in red, significant correlations are written in bold letters. (CFMT, final score; Feat, sensitivity to featural changes in a face; Conf, sensitivity to configural changes in a face; Shells, Faces, Blue objects: d′ *values for shells, faces and the blue objects in the object recognition task*.)

Performance on all four face-related tasks [CFMT, sensitivity to features (Feat) and configuration (Conf), object recognition task with face stimuli (Faces)] were positively and significantly correlated or approached significance. The effect sizes of these correlations (0.22 < *r* < 0.49) were medium and hence the proportions of shared variance (0.05 < *r*^2^ < 0.24) were rather small. Thus, we assume that although different aspects of face perception are investigated by the tests (i.e., recognition performance, memory, and sensitivity to features and configuration) these aspects are nevertheless to some degree dependent from each other.

Surprisingly there was another significant, but negative correlation (with a rather small effect size): participants with a high sensitivity to configuration of a face tended to have bad performance in the shell recognition task. The small proportion of shared variance of *r*^2^ = 0.09 led us to refrain from any speculations.

## General discussion

The combination of tasks used in this study tested various aspects of face and object recognition, which allowed us to compare directly the influence of CP and the ORE. Our hypothesis, based on previous findings, was that in CP and the ORE the same underlying mechanisms might be affected. While we could disprove this hypothesis (this is discussed in detail below), we were able to confirm results of previous studies and importantly we gain new insights concerning the similarities between these two impairments of face recognition.

First, we were able to replicate the findings that congenital prosopagnosics exhibit face recognition deficits but no object recognition deficits (Le Grand et al., 2006). Second, we were able to replicate the ORE with our Koreans in the CFMT. Interestingly our results differ somewhat from the results by McKone et al. (2012) who only found a trend toward a different performance between their Asian and Caucasian participants on the original CFMT. A possible explanation for this discrepancy is that their Asian participants may have had more experience with Caucasian faces because they were overseas students living in Australia at the time of testing. Our Asian participants were tested in Korea and thus were likely to have less experience with Caucasian faces. Third, our experiment testing sensitivity toward featural and configural changes within a face resolves discrepancies between studies testing sensitivity toward featural and configural facial information for prosopagnosics (Le Grand et al., 2006; Yovel and Duchaine, 2006). Our results, in the context of previous studies, show that, compared with German controls, prosopagnosics exhibit an impaired sensitivity toward configural information and possibly and only to a lesser extent, toward featural information of a face.

Importantly, besides those confirmations of previous findings, we report the new finding that sensitivities to features and configuration of a face differ between Korean and prosopagnosic participants. For both groups, the observed sensitivity to the featural changes in a face was about the same. The Koreans, however, were better than prosopagnosics (and as good as Germans) at detecting fine changes in configural information in a face. When comparing CP with the ORE, we asked if they derive from a disturbance in the same underlying mechanisms. Our results indicate that this is not the case: especially the difference in absolute sensitivity to configural and featural changes for prosopagnosic and other-race observers is a strong indicator that CP and the ORE impair face recognition differently. As we used the same face stimuli to test all participant groups, our results indicate that lacking expertise for a certain face group does not impact configural processing of those faces (Korean group), while CP does (prosopagnosic group). Even though we cannot explain what exactly causes this difference, these results clearly show that there are different mechanisms underlying both impairments. Therefore, we are not “prosopagnosic for other-race faces” (see also Wang et al., 2009).

Our second main finding is that face recognition performance is more strongly affected by CP than by the ORE. Our prosopagnosics performed significantly worse than the Koreans in all face recognition tasks. A possible explanation is that generally an existing expertise for same-race faces can be used for recognition of untrained other-race faces, while no such expertise exists in CP (Carbon et al., 2007).

The findings of our test battery also have some further implications for the general understanding of face perception and face processing. First, we find that better configural sensitivity relates to better face recognition ability. Koreans and Germans performed significantly better in the general face recognition task Cambridge Face Memory Test, and at the same time showed a significantly higher sensitivity to configural changes in our second test than the prosopagnosics. This importance of configural processing for holistic processing was so far only shown by disrupting configural information, e.g., by the inversion effect (Freire et al., 2000). Our finding is an important result that allows us to get further insight about which aspect of face recognition relates with being a good face recognizer. When correlating performance in the CFMT with the sensitivity to configural changes across all participants, we obtained a significant but medium proportion of shared variance of *r*^2^ = 0.24 (which is larger than the proportion of shared variance of *r*^2^ = 0.09 of performance in the CFMT and sensitivity to featural changes). Until now studies looking for processes related to face recognition performance mostly correlated it to holistic processing in general (e.g., performance in the composite face task or part-whole-face-task). Different proportions of shared variance were found: either zero (*r*^2^ = 0.003, Konar et al., 2010), or medium (*r*^2^ = 0.16, Richler et al., 2011), or similar to our value (*r*^2^ = 0.21, DeGutis et al., 2013). The range of results in these studies might be explained by the different measures used for face recognition (CFMT vs. own identity recognition tasks), holistic processing (composite face task vs. part-whole-face-task) and different approaches to calculate the effect scores (subtraction scores vs. regression scores, and partial vs. complete composite face design). Whether general problems in processing faces results in an inability to see subtle differences in facial configuration, whether a reduced sensitivity to configuration results in impaired face recognition ability, or whether configural sensitivity and face recognition performance are impaired by disrupting a common underlying process remains an open question. This is a decade-old, and as-of-yet unanswered issue (Barton et al., 2003) which we cannot address using our current data. Nevertheless, our results strengthen the hypothesis that configural processing is linked to face recognition ability, but the proportions of shared variance are only low to medium, which show that configural sensitivity and/or holistic processing cannot solely explain face processing abilities.

The second implication of our findings for face processing stems from the fact that we find no difference in terms of sensitivity to facial features between Koreans and prosopagnosics. This suggests that this aspect is not crucial for determining face recognition abilities. This finding is supported by the low effect size found in correlating the sensitivity to featural changes with face recognition performance (tested either using the CFMT or the face recognition performance in the object recognition task): only a small portion of the variance of face recognition abilities is explained by the sensitivity to differences in features (*r*^2^ = 0.09 and 0.11 in both cases).

Overall, with our test battery we were able to replicate results of previous studies and provide new insights into the face processing disturbances caused by CP and the ORE. Thus, when a (Caucasian) prosopagnosic person tries to explain his or her condition to a (Korean) non-prosopagnosic person with the ORE (“They all look the same to you; everyone else does for me, too”) this is an inexact comparison. Although the perception of Caucasian faces by Koreans and prosopagnosics observers differs, the analogy probably gives at least an idea of the problems congenital prosopagnosics (though to a stronger extent) have to face.

### Conflict of interest statement

The authors declare that the research was conducted in the absence of any commercial or financial relationships that could be construed as a potential conflict of interest.

## Footnotes

^1^Only pixels which actually differed between both images were taken into consideration. Thus, the gray background and the common outer face shape were omitted for the averaging process. This avoids an artificial reduction of the mean pixel distances.

## References

[B1] Avidan G. Behrmann M. (2009). Functional MRI reveals compromised neural integrity of the face processing network in congenital prosopagnosia. Curr. Biol. 19, 1146–1150 10.1016/j.cub.2009.04.060 19481456 PMC2711224

[B2] Avidan G. Hasson U. Malach R. Behrmann M. (2005). Detailed exploration of face-related processing in congenital prosopagnosia: 2. Functional neuroimaging findings. J. Cogn. Neurosci. 17, 1150–1167 10.1162/0898929054475145 16102242

[B3] Avidan G. Tanzer M. Behrmann M. (2011). Impaired holistic processing in congenital prosopagnosia. Neuropsychologia 49, 2541–2552 10.1016/j.neuropsychologia.2011.05.002 21601583 PMC3137703

[B4] Avidan G. Thomas C. Behrmann M. (2008). An integrative approach towards understanding the psychological and neural basis of congenital prosopagnosia, in Cortical Mechanisms of Vision, eds Jenkin M. Harris L. R. (New York, NY: Cambridge University Press), 241–270 Available online at: http://tdlc.ucsd.edu/research/publications/Avidan_Integrative_Approach_2009.pdf (Accessed June 19, 2014).

[B5] Barton J. J. S. Cherkasova M. V. Press D. Z. Intriligator J. M. O'Connor M. (2003). Developmental prosopagnosia: astudy of three patients. Brain Cogn. 51, 12–30 10.1016/S0278-2626(02)00516-X 12633587

[B6] Behrmann M. Avidan G. Marotta J. J. Kimchi R. (2005). Detailed exploration of face-related processing in congenital prosopagnosia: 1. Behavioral findings. J. Cogn. Neurosci. 17, 1130–1149 10.1162/0898929054475154 16102241

[B7] Bernstein M. J. Young S. G. Hugenberg K. (2007). The cross-category effect: mere social categorization is sufficient to elicit an own-group bias in face recognition. Psychol. Sci. 18, 706–712 10.1111/j.1467-9280.2007.01964.x 17680942

[B8] Blais C. Jack R. E. Scheepers C. Fiset D. Caldara R. (2008). Culture shapes how we look at faces. PLoS ONE 3:e3022 10.1371/journal.pone.0003022 18714387 PMC2515341

[B9] Carbon C.-C. Grüter T. Weber J. E. Lueschow A. (2007). Faces as objects of non-expertise: processing of thatcherised faces in congenital prosopagnosia. Perception 36, 1635–1645 10.1068/p5467 18265844

[B10] Collishaw S. M. Hole G. J. (2000). Featural and configurational processes in the recognition of faces of different familiarity. Perception 29, 893–909 10.1068/p2949 11145082

[B11] DeGutis J. M. Bentin S. Robertson L. C. D'Esposito M. (2007). Functional plasticity in ventral temporal cortex following cognitive rehabilitation of a congenital prosopagnosic. J. Cogn. Neurosci. 19, 1790–1802 10.1162/jocn.2007.19.11.1790 17958482

[B12] DeGutis J. M. Wilmer J. Mercado R. J. Cohan S. (2013). Using regression to measure holistic face processing reveals a strong link with face recognition ability. Cognition 126, 87–100 10.1016/j.cognition.2012.09.004 23084178

[B13] Duchaine B. C. Nakayama K. (2005). Dissociations of face and object recognition in developmental prosopagnosia. J. Cogn. Neurosci. 17, 249–261 10.1162/0898929053124857 15811237

[B14] Duchaine B. C. Nakayama K. (2006). The Cambridge face memory test: results for neurologically intact individuals and an investigation of its validity using inverted face stimuli and prosopagnosic participants. Neuropsychologia 44, 576–585 10.1016/j.neuropsychologia.2005.07.001 16169565

[B15] Duchaine B. C. Yovel G. Nakayama K. (2007). No global processing deficit in the Navon task in 14 developmental prosopagnosics. Soc. Cogn. Affect. Neurosci. 2, 104–113 10.1093/scan/nsm003 18985129 PMC2555457

[B16] Esins J. Bülthoff I. Schultz J. (2011). The role of featural and configural information for perceived similarity between faces. J. Vis. 11:673 10.1167/11.11.673

[B17] Fowler D. R. Meinhardt H. Prusinkiewicz P. (1992). Modeling seashells. ACM SIGGRAPH Comput. Grap. 26, 379–387 10.1145/133994.134096

[B18] Freire A. Lee K. Symons L. A. (2000). The face-inversion effect as a deficit in the encoding of configural information: direct evidence. Perception 29, 159–170 10.1068/p3012 10820599

[B19] Gaißert N. Wallraven C. Bülthoff H. H. (2010). Visual and haptic perceptual spaces show high similarity in humans. J. Vis. 10, 1–20 10.1167/10.11.2 20884497

[B20] Goffaux V. Hault B. Michel C. Vuong Q. C. Rossion B. (2005). The respective role of low and high spatial frequencies in supporting configural and featural processing of faces. Perception 34, 77–86 10.1068/p5370 15773608

[B21] Grüter T. Grüter M. Carbon C.-C. (2008). Neural and genetic foundations of face recognition and prosopagnosia. J. Neuropsychol. 2, 79–97 10.1348/174866407X231001 19334306

[B22] Hayward W. G. Rhodes G. Schwaninger A. (2008). An own-race advantage for components as well as configurations in face recognition. Cognition 106, 1017–1027 10.1016/j.cognition.2007.04.002 17524388

[B23] Hugenberg K. Young S. G. Bernstein M. J. Sacco D. F. (2010). The categorization-individuation model: an integrative account of the other-race recognition deficit. Psychol. Rev. 117, 1168–1187 10.1037/a0020463 20822290

[B24] Kennerknecht I. Ho N. Y. Wong V. C. N. (2008). Prevalence of hereditary prosopagnosia (HPA) in Hong Kong Chinese population. Am. J. Med. Genet. A 146A, 2863–2870 10.1002/ajmg.a.32552 18925678

[B25] Kimchi R. Behrmann M. Avidan G. Amishav R. (2012). Perceptual separability of featural and configural information in congenital prosopagnosia. Cogn. Neuropsychol. 29, 447–463 10.1080/02643294.2012.752723 23428081

[B26] Konar Y. Bennett P. J. Sekuler A. B. (2010). Holistic processing is not correlated with face-identification accuracy. Psychol. Sci. 21, 38–43 10.1177/0956797609356508 20424020

[B27] Kress T. Daum I. (2003). Developmental prosopagnosia: a review. Behav. Neurol. 14, 109–121 10.1155/2003/520476 14757987 PMC5497561

[B28] Le Grand R. Cooper P. A. Mondloch C. J. Lewis T. L. Sagiv N. De Gelder B. (2006). What aspects of face processing are impaired in developmental prosopagnosia? Brain Cogn. 61, 139–158 10.1016/j.bandc.2005.11.005 16466839

[B29] Lobmaier J. S. Bölte J. Mast F. W. Dobel C. (2010). Configural and featural processing in humans with congenital prosopagnosia. Adv. Cogn. Psychol. 6, 23–34 10.2478/v10053-008-0074-4 20689639 PMC2916664

[B30] Macmillan N. A. Creelman C. D. (2005). Detection Theory: A User's Guide. 2nd Edn. Mahwah, NJ: Lawrence Erlbaum Associates

[B31] Maurer D. Le Grand R. Mondloch C. J. (2002). The many faces of configural processing. Trends Cogn. Sci. 6, 255–260 10.1016/S1364-6613(02)01903-4 12039607

[B32] Maurer D. O'Craven K. M. Le Grand R. Mondloch C. J. Springer M. V. Lewis T. L. (2007). Neural correlates of processing facial identity based on features versus their spacing. Neuropsychologia 45, 1438–1451 10.1016/j.neuropsychologia.2006.11.016 17204295

[B33] McKone E. Aimola Davies A. Fernando D. Aalders R. Leung H. Wickramariyaratne T. (2010). Asia has the global advantage: race and visual attention. Vision Res. 50, 1540–1549 10.1016/j.visres.2010.05.010 20488198

[B34] McKone E. Brewer J. L. MacPherson S. Rhodes G. Hayward W. G. (2007). Familiar other-race faces show normal holistic processing and are robust to perceptual stress. Perception 36, 224–248 10.1068/p5499 17402665

[B35] McKone E. Stokes S. Liu J. Cohan S. Fiorentini C. Pidcock M. (2012). A robust method of measuring other-race and other-ethnicity effects: the Cambridge face memory test format. PLoS ONE 7: e47956 10.1371/journal.pone.0047956 23118912 PMC3484147

[B36] Meissner C. A. Brigham J. C. (2001). Thirty years of investigating the own-race bias in memory for faces: a meta-analytic review. Psychol. Pub. Policy Law 7, 3–35 10.1037/1076-8971.7.1.3

[B37] Michel C. Rossion B. Han J. Chung C.-S. Caldara R. (2006). Holistic processing is finely tuned for faces of one's own race. Psychol. Sci. 17, 608–615 10.1111/j.1467-9280.2006.01752.x 16866747

[B38] Mondloch C. J. Elms N. Maurer D. Rhodes G. Hayward W. G. Tanaka J. W. (2010). Processes underlying the cross-race effect: an investigation of holistic, featural, and relational processing of own-race versus other-race faces. Perception 39, 1065–1085 10.1068/p6608 20942358

[B39] Rhodes G. Brake S. Taylor K. Tan S. (1989). Expertise and configural coding in face recognition. Br. J. Psychol. 80, 313–331 10.1111/j.2044-8295.1989.tb02323.x 2790391

[B40] Rhodes G. Hayward W. G. Winkler C. (2006). Expert face coding: configural and component coding of own-race and other-race faces. Psychon. Bull. Rev. 13, 499–505 10.3758/BF03193876 17048737

[B41] Richler J. J. Cheung O. S. Gauthier I. (2011). Holistic processing predicts face recognition. Psychol. Sci. 22, 464–471 10.1177/0956797611401753 21393576 PMC3077885

[B42] Rivolta D. Palermo R. Schmalzl L. Coltheart M. (2011). Covert face recognition in congenital prosopagnosia: a group study. Cortex 48, 1–9 10.1016/j.cortex.2011.01.005 21329915

[B43] Rotshtein P. Geng J. J. Driver J. Dolan R. J. (2007). Role of features and second-order spatial relations in face discrimination, face recognition, and individual face skills: behavioral and functional magnetic resonance imaging data. J. Cogn. Neurosci. 19, 1435–1452 10.1162/jocn.2007.19.9.1435 17714006 PMC2600425

[B44] Rushton J. P. Jensen A. R. (2005). Thirty years of research on race differences in cognitive ability. Psychol. Pub. Policy Law 11, 235–294 10.1037/1076-8971.11.2.235

[B45] Stollhoff R. Jost J. Elze T. Kennerknecht I. (2011). Deficits in long-term recognition memory reveal dissociated subtypes in congenital prosopagnosia. PLoS ONE 6:e15702 10.1371/journal.pone.0015702 21283572 PMC3026793

[B46] Towler J. Gosling A. Duchaine B. C. Eimer M. (2012). The face-sensitive N170 component in developmental prosopagnosia. Neuropsychologia 50, 3588–3599 10.1016/j.neuropsychologia.2012.10.017 23092937

[B47] Troje N. F. Bülthoff H. H. (1996). Face recognition under varying poses: the role of texture and shape. Vision Res. 36, 1761–1771 10.1016/0042-6989(95)00230-8 8759445

[B48] Vetter T. Blanz V. (1999). A morphable model for the synthesis of 3D faces, in SIGGRAPH'99 Proceedings of the 26th annual conference on Computer graphics and interactive techniques (New York, NY: ACM Press/Addison-Wesley Publishing Co.), 187–194 10.1145/311535.311556

[B49] Wang H. Stollhoff R. Elze T. Jost J. Kennerknecht I. (2009). Are we all prosopagnosics for other race faces? Perception 38 ECVP Abstract Supplement, 78 10.1371/journal.pone.0003022 21570991 PMC11025337

[B50] Yovel G. Duchaine B. C. (2006). Specialized face perception mechanisms extract both part and spacing information: evidence from developmental prosopagnosia. J. Cogn. Neurosci. 18, 580–593 10.1162/jocn.2006.18.4.580 16768361

[B51] Yovel G. Kanwisher N. (2004). Face perception: domain specific, not process specific. Neuron 44, 889–898 10.1016/j.neuron.2004.11.018 15572118

