# Corrigendum: Do congenital prosopagnosia and the other-race effect affect the same face recognition mechanisms?

**DOI:** 10.3389/fnhum.2015.00294

**Published:** 2015-05-27

**Authors:** Janina Esins, Johannes Schultz, Claudia Stemper, Ingo Kennerknecht, Christian Wallraven, Isabelle Bülthoff

**Affiliations:** ^1^Department of Human Perception, Cognition and Action, Max Planck Institute for Biological CyberneticsTübingen, Germany; ^2^Department of Psychology, Durham UniversityDurham, UK; ^3^Institute of Human Genetics, Westfälische Wilhelms-Universität MünsterMünster, Germany; ^4^Department of Brain and Cognitive Engineering, Korea UniversitySeoul, South Korea

**Keywords:** congenital prosopagnosia, other-race effect, face recognition, Asian, Caucasian

The list of authors of this article should be corrected in order to include Ms. Claudia Stemper and Prof. Dr. Ingo Kennerknecht. Both authors have contributed significantly to this study. Almost all prosopagnosic subjects were ascertained by them. Therefore, the authorship order should be considered as follows:

Janina Esins^1*^, Johannes Schultz^1,2^, Claudia Stemper^3^, Ingo Kennerknecht^3^, Christian Wallraven^4^ and Isabelle Bülthoff^1,4^

^1^ Department of Human Perception, Cognition and Action, Max Planck Institute for Biological Cybernetics, Tübingen, Germany
^2^ Department of Psychology, Durham University, Durham, UK
^3^ Institute of Human Genetics, Westfälische Wilhelms-Universität Münster, Münster, Germany
^4^ Department of Brain and Cognitive Engineering, Korea University, Seoul, South Korea

The authors regret the error.

## Conflict of interest statement

The authors declare that the research was conducted in the absence of any commercial or financial relationships that could be construed as a potential conflict of interest.

