# Supplementary material for: Do congenital prosopagnosia and the other-race effect affect the same face recognition mechanisms?
Source: Front Hum Neurosci. 2014 Sep 29;8:759. doi: 10.3389/fnhum.2014.00759 (PMC4179381; doi:10.3389/fnhum.2014.00759)
Supplement: Supplementary file 1 [file DataSheet1.DOCX]

|  | | Statistical test results | |
| --- | --- | --- | --- |
|  |  | All 21 participants per group | 17 participants per group  (w/o the suspicious 4 Korean and their matched Ger and cPA participants) |
| CFMT | one-way-ANOVA | one-way-ANOVA  F(2,62) = 67.34,  p < 0.001, η_p_^2^ = 0.69  Tukey HSD post hoc tests  CP vs KO: p ≤ 0.002  CP vs GE: p ≤ 0.002  KO vs GE: p = 0.002 | one-way-ANOVA  F(2,50) = 68.1667,  p < 0.001, η_p_^2^ = 0.7396  Tukey HSD post hoc tests  CP vs KO: p ≤ 0.001  CP vs GE: p ≤ 0.001  KO vs GE: p = 0.094 |
| Sensitivity to features and configuration  (continued) | 2x3 ANOVA on regression slopes | Sign. main effect of change type (configural, featural) (F(1,60)=233.7, p<0.001, η^2^=0.46, η_p_^2^=0.796  Sign. main effect of participant group (German, Korean, prosopagnosic)  (F(2,60)=6.46, p=0.003, η^2^=0.07, η_p_^2^=0.18  Sign. interaction between change type and participant group  (F(2,60)=5.48, p=0.007, η^2^=0.02, η_p_^2^=0.15 | Sign. main effect of change type (configural, featural) (F(1,48)=198.54, p<0.001, η_p_^2^=0.805  Sign. main effect of participant group (German, Korean, prosopagnosic)  (F(2,48)=5.54, p=0.007,  η_p_^2^=0.19  Sign. interaction between change type and participant group  (F(2,48)=5.49, p=0.007,  η_p_^2^=0.19 |
|  | one-way-ANOVA of simple effect change type (group differences on each change type (configural, featural)) | Features:  one-way-ANOVA  F(2,62)=3.12, p=0.0515, η_p_^2^=0.09  Tukey HSD post hoc,  CP vs GE: p=0.051, both other differences p>0.17  Configuration:  one-way-ANOVA  F(2,62)=9.11, p<0.001, η_p_^2^=0.23  Tukey HSD post hoc,  CP vs GE: p<0.003,  CP vs KO: p<0.003  GE vs KO: non-sign. | Features:  one-way-ANOVA  F(2,50)=1.45, p=0.24,  η_p_^2^=0.06  Tukey HSD post hoc,  CP vs GE: p=0.21, both other differences p>0.67  Configuration:  one-way-ANOVA  F(2,50)=8.7, p<0.001, η_p_^2^=0.27  Tukey HSD post hoc,  CP vs GE: p=0.011,  CP vs KO: p=0.001  GE vs KO: p=0.602 |
| (continued)  Sensitivity to features and configuration | one-way ANOVA on featural advantageFFigure Figure 7: | one-way ANOVA  F(2,62)=5.48, p=0.007, η_p_^2^=0.15  Tukey HSD post hoc  CP vs. KO: p=0.005  KO vs. GE: p=0.091  CP vs. GE: p=0.51 | one-way ANOVA  F(2,50)=5.49, p=0.007, η_p_^2^=0.19  Tukey HSD post hoc  CP vs. KO: p=0.005  KO vs. GE: p=0.197  CP vs. GE: p=0.273 |
| Object recognition | 3x3 ANOVA on d' | main effect of participant group  F(2,60)=1.22, p=0.303, η_p_^2^=0.04  Sign. main effect of object category  F(2,60)=145.54, p<0.001, η_p_^2^=0.71  Sign. interaction between participant group and object category  F(4,120)=7.14, p<0.001, η_p_^2^=0.19 | main effect of participant group  F(2,48)=0.76, p=0.473  Sign. main effect of object category  F(2,48)=119.19, p<0.001,  Sign. interaction between participant group and object category  F(4,96)=4.35, p+0.003, |
|  | A one-way-ANOVA of simple effect object type  (group differences on each object type (configural, featural)) | Faces:  one-way-ANOVA  F(2,62)=8.14, p=0.001, η_p_^2^=0.04  Games Howel post hoc  CP vs KO: p≤0.01  CP vs GE: p≤0.01  GE vs KO: p>0.2  Shells and Greebles:  one-way-ANOVAs  p>0.2  Games Howel post hoc  all comparisons: p>0.2 | Faces:  one-way-ANOVA  F(2,50)=5.063, p=0.01  Games Howel post hoc  CP vs KO: p=0.024  CP vs GE: p=0.024  GE vs KO: p=0.974  Shells and Greebles:  one-way-ANOVAs  Shell: p>0.45  Games Howel post hoc  all comparisons: p>0.16 |
|  |  |  |  |
